# Formation of a New Entity to Support Effective Use of Technology in Medical Education: The Student Technology Committee

**DOI:** 10.2196/mededu.4676

**Published:** 2015-09-17

**Authors:** Jared Andrew Shenson, Ryan Christopher Adams, S. Toufeeq Ahmed, Anderson Spickard

**Affiliations:** ^1^School of MedicineVanderbilt UniversityNashville, TNUnited States; ^2^Department of Biomedical InformaticsVanderbilt University Medical CenterNashville, TNUnited States; ^3^Department of MedicineVanderbilt University Medical CenterNashville, TNUnited States

**Keywords:** committee membership, educational technology, medical education, medical students, organizational innovation, organizational models

## Abstract

**Background:**

As technology in medical education expands from teaching tool to crucial component of curricular programming, new demands arise to innovate and optimize educational technology. While the expectations of today’s digital native students are significant, their experience and unique insights breed new opportunities to involve them as stakeholders in tackling educational technology challenges.

**Objective:**

The objective of this paper is to present our experience with a novel medical student-led and faculty-supported technology committee that was developed at Vanderbilt University School of Medicine to harness students’ valuable input in a comprehensive fashion. Key lessons learned through the initial successes and challenges of implementing our model are also discussed.

**Methods:**

A committee was established with cooperation of school administration, a faculty advisor with experience launching educational technologies, and a group of students passionate about this domain. Committee membership is sustained through annual selective recruitment of interested students.

**Results:**

The committee serves 4 key functions: acting as liaisons between students and administration; advising development of institutional educational technologies; developing, piloting, and assessing new student-led educational technologies; and promoting biomedical and educational informatics within the school community. Participating students develop personally and professionally, contribute to program implementation, and extend the field’s understanding by pursuing research initiatives. The institution benefits from rapid improvements to educational technologies that meet students’ needs and enhance learning opportunities. Students and the institution also gain from fostering a campus culture of awareness and innovation in informatics and medical education. The committee’s success hinges on member composition, school leadership buy-in, active involvement in institutional activities, and support for committee initiatives.

**Conclusions:**

Students should have an integral role in advancing medical education technology to improve training for 21st-century physicians. The student technology committee model provides a framework for this integration, can be readily implemented at other institutions, and creates immediate value for students, faculty, information technology staff, and the school community.

## Introduction

### Background

Over the last 15 years, technology has become pervasive in medical education training at both the undergraduate and graduate levels [1,2]. The excitement to adopt this technology is often attributed to the unique and evolving needs and capabilities of today’s digital native learners [3,4]. Indeed, medical students and residents are eager to integrate digital resources into their training [5-7]. Technology is no longer only a teaching tool, but also a crucial component of curricular programming. Many studies have offered goals for new instructional technologies [4,8], describing the need to “transform learning into a more collaborative, personalized, and empowering experience that can inspire a new generation of learners.” [1] With the explosion of new information, new instructional modalities, and the accelerating pace of technological evolution, there has never been a more opportune time to innovate in medical education technology. There are significant challenges, however, for educators and information technology (IT) professionals as they pursue this agenda, including the rapid evolution of technologies [2], highly heterogeneous learners [9,10], diverse educational pedagogies [1], limited funding [11], and varying levels of institutional support [11-13].

Success in overcoming these barriers depends on a systematic approach to project development, including the critical need for learner involvement throughout the process [9,11,14]. Medical students can shape technology development and integration efforts because they better understand student culture and goals, are often more comfortable with IT than faculty, and can offer creative ideas outside of traditional approaches [15-17]. Despite these calls for student involvement, only 21% of United States and Canadian medical schools surveyed sought student input on new applications and services, and even fewer engaged students in student-led contributions to educational technologies [13]. More commonly, student input is solicited only after technology implementation via satisfaction surveys [2,8]. Review of the academic and lay literature identified no reports of formalized or longitudinal student involvement in medical education technology.

The few reports that describe student input in integrating educational technology confirm the vital role students play in this work and their ability to advance the field by novel research projects. Students have worked with faculty in developing a Web-based student resource portal [15], in revising Web-based teaching modules for their peers [17], and in supporting iPad integration into the preclinical curriculum [18]. Other students have worked independently of institutional support to build and pilot novel collaborative studying tools [19], and to test mobile resources for the clinical learner [20-22].

### Objective

The rising tide of interest in and opportunities for medical education technologies, combined with the clear successes of student involvement in the aforementioned examples, prompted us to reconsider how motivated students could become engaged stakeholders who could inform the effective use of these innovations. Determining how to harness students’ valuable input in a systemic fashion was integral to our approach. In this paper, we describe the ideation, implementation, and impact of a novel medical student-led technology committee, examine its unique benefits, and discuss keys to success for adopting this model at other institutions.

## Methods

### Implementing a Student Technology Committee

At our institution in the summer of 2012, student input regarding educational technologies was highly fragmented, leading to student frustration with the state of technology offerings. Born from the opportunity to help shape the future, a student interest group formed organically to focus on driving improvements in educational technologies from the student perspective. Over the 1st year, the group undertook multiple projects, including updates to the learning management system (LMS), a pilot study of iPad use in the gross anatomy laboratory, and a clinical podcast series. Strong relationships between senior group members and key faculty were essential to early work. These contacts provided insight into institutional priorities, made connections with relevant faculty and IT staff, and supported group initiatives financially and intellectually. Faculty and staff were appreciative of the high-quality input provided by the group and the efforts of its members and applauded the group’s educational technology research endeavors.

At the end of the 1st year, the group’s efforts were formalized as the “student technology committee” (STC). The Assistant Dean of Educational Informatics and Technology was selected as faculty advisor to provide close collaboration between the STC and institutional leadership. STC presidents and the faculty advisor enumerated core competencies and operating principles for the committee (Table 1), which were modeled after student-led curriculum committees [23,24].

**Table 1:** Summary of the student technology committee bylaws.

Bylaws article	Details
Goals and purpose	Four core goals spanning medical school curriculum (see Areas 1-4 in the “Results” section)
Composition	Two to three student representatives per class year
	Faculty advisor is specified (eg, Assistant Dean of Educational Informatics and Technology)
Membership term	Members serve throughout their 4 years of medical school
	Membership may be surrendered voluntarily or revoked for failure of responsibilities or conduct
	Affiliate member status granted to students on temporary leave from medical school (eg, research year, second degree)
Elections	First-year students apply via written application with secondary interviews
	New members selected based on holistic review
	Elections in other class years held as needed if fewer than 2 representatives for a given year or a member leaves the committee
Leadership	President or Co-Presidents (2) serve 1-year terms (re-election allowed)
	President(s) elected by popular vote of committee members
	Serve as liaisons between committee and school leadership, faculty, staff, and other student committees
Meetings	Held 2 times/month on alternating weeks, additional meetings as needed
	All meeting minutes recorded and archived
	First meeting: includes faculty advisor; open to all faculty and staff seeking student technology committee input; focus on project updates as well as administrative and faculty priorities
	Second meeting: closed; focus on brainstorming new project ideas, addressing current challenges in project execution
Budget	Annual budget prepared by committee president(s) with approval by committee and faculty advisor
	Funds secured from institutional student group grants and the Office of Educational Informatics and Technology

### Student Membership and Project Operations

Given the unique educational environment of each class year, the STC includes representatives from across the student body. Members serve on the committee throughout their medical school experience to promote continuity and commitment, especially in consideration of the many longitudinal projects the committee undertakes. The committee president(s) is elected annually. Senior (3rd- or 4th-year) students typically will be selected for this position on the basis of availability and flexibility in their schedules as well as experience working with the committee.

New members are selected from the 1st-year class via an application process soon after matriculation. Applicants respond to questions concerning interest in the committee, prior experience with technology, ideas for new technology in the medical school, and thoughts about the future of medical technology. These questions assess applicants’ motivation for involvement, relevant experience and knowledge, and ability to think creatively about problems the committee may address. Top candidates, identified based on written applications, are interviewed to evaluate for personal characteristics we have identified to be present in our most successful members (Table 2). Final selections are made based on a holistic review, including consideration as to how applicants’ skills may complement those of existing and new members.

The committee operates on a dynamic project management model wherein a single member is appointed the lead for a given project and may flexibly recruit and release additional members to support that project as needed. When serving as project leads, junior members are frequently mentored by senior members in areas including project management, research methods, and administrative and staff contacts. Accountability for project deliverables is reinforced through monthly goal setting and project review, which occur as part of the committee’s twice-monthly meetings (Table 1).

**Table 2:** Qualities of successful student technology committee members.

Quality	How it supports success
Passionate	Ensures baseline understanding and interest in medical education pedagogy, educational technology, and/or medical practice technology
	Drives personal investment in committee work despite multiple demands on available time
Creative	Supports flexible problem solving in projects with high complexity and multiple resource constraints
	Enables out-of-the-box thinking, creating opportunity for true innovation
Problem solver	Identifies limits of personal knowledge and when to seek help
	Improves self-sufficiency on personal projects
	In tandem with creativity, enhances committee efforts to address challenging projects
Self-starting	Minimizes required project oversight
	Improves efficiency of project execution
Strong communication	Enables effective relationships spanning students, staff, and faculty
	Aids in team understanding of individual’s goals, methods, challenges, and results
Cooperative	Fosters personal connections with other members
	Supports work of all members to advance committee goals
	Promotes positive committee culture to improve morale and member effectiveness
Technical understanding	Familiarity with major consumer technologies (eg, Web services, mobile phones, tablets, wearables) and core technical topics (eg, servers, Wi-Fi)

## Results

### Delivering on Our Core Work Areas

#### Area 1: Serve as Liaisons Between Students and Administration, IT Staff, and Course Directors With Regard to All Technologies Related to the Curriculum

Committee members’ close proximity to the student body’s day-to-day challenges yields an ideal communication channel between students, faculty, and staff. The committee regularly solicits student feedback regarding institutional technologies, while also receiving unsolicited feedback in person, by email, or via anonymous online forms. Issues are communicated directly to the most relevant point person, thereby supporting efficient resolution of student concerns and minimizing communication hurdles between IT staff and students. The committee also works to share the value of new technologies with the student body and to keep them apprised of improvements that are the direct result of their input, closing the feedback loop.

Following the launch of our institution’s new LMS, the STC helped to facilitate rapid-cycle development to eliminate software bugs and address essential student needs. Building on first-hand experience using the LMS daily, committee members collected data from in-person discussion with peers and through an online survey. In just 2 weeks, the committee generated reports from end-user feedback that directly informed administrative planning for short- and long-term platform improvements.

#### Area 2: Serve as a Student Advisory Panel for Continuous Development and Improvement of Technology Involved in the Curriculum

The STC provides faculty and staff with easy, direct access to a mixed cohort of students for soliciting input at any phase of the development process. To date, the student committee has provided input on more than a dozen initiatives and projects, including the LMS, the online learning portfolio, student Web dashboards, a mobile real-time feedback tool [25], the School of Medicine website, iPads and TVs in the gross anatomy laboratory, and wireless printing in the computer laboratory. Faculties have consistently found the STC input on various projects to be extremely useful, lending new insights to guide future directions.

#### Area 3: Develop, Pilot, and Assess New Uses of Educational Technology or Instruction Related to Technology

As curriculum consumers, students are poised to identify numerous opportunities for novel use of instructional technology. The STC seeks to foster a research and development environment in which members, with suitable mentorship from senior student peers and faculty, can explore novel uses of educational technology. The school supports this mission by providing financial support for pilot projects and dissemination of lessons learned through journal publications and conference presentations. Academic informatics and/or computer science departments can also play an important role in supporting committee research endeavors.

A student-led pilot study and subsequent class-wide integration of iPads into our institution’s gross anatomy laboratory exemplifies the extent to which the STC model can impact a school’s education ecology. Building on the experiences of other institutions [18,26], committee members rapidly implemented a low-cost pilot study to test iPads loaded with two-dimensional and three-dimensional anatomy atlases with 60 students over a 2-month period. Poststudy surveys revealed that students found it to be a positive addition to their dissection experience with distinct educational value; however, they also identified key shortcomings to address. Working with school leadership, the STC used this information to advocate successfully for deployment of iPads at all tables in the anatomy laboratory. Since then, the STC conducts ongoing analysis to help administrators tailor the hardware and software to student needs.

#### Area 4: Promote Informatics, Consumer Technology, and Workplace Technology, and Their Applications in Medicine and Education at Our Institution and Abroad

The STC aims to prepare medical students to work in the rapidly evolving health care system under the heavy influence of technological advances. Explicit training in this area at our institution has been scarce. The committee has identified unique opportunities to provide and facilitate instruction concerning effective use of educational technology and to inform student understanding and dialogue about the technologies shaping 21st-century health care. Committee members created online resource guides to aid students in getting the most out of their laptops and mobile devices as learning and clinical tools. The STC developed a “Tech Talks” series of lunch-hour faculty lectures to engage students and the medical center community in thinking critically about popular, clinically relevant topics in biomedical informatics and medical technologies. In addition, efforts are underway to develop elective seminar courses teaching medical students about the changing dynamics of the patient-physician relationship as altered by consumer and medical technologies.

### Benefitting Students and the Institution

Formalizing student involvement in educational technology using the STC model offers clear benefits to the school as well as committee members. Numerous advancements to the educational ecology, including existing and new technologies, are possible (as outlined in the previous section), benefitting all students in the institution through potential for greater personalization of learning as well as improved satisfaction and performance. Working toward a shared goal of improving the academic experience for medical students, the committee, together with faculty and staff, develops a collaborative and constructive relationship that enhances the work of all parties. The IT staff are readily able to engage in rapid-cycle feedback and development without significant time and energy investment, increasing the likelihood of project success. Core curricular faculty and administration gain direct insight into student priorities, wants, and needs, which may differ substantially from thoughts and plans developed without such input. They also learn to break down traditional assumptions about the limited roles medical students should play in institutional initiatives [16]. In some cases, the STC may also assume significant responsibility for development and/or testing of new technologies, reducing the expenditure of resources by the institution on those efforts.

Student members of the STC grow personally and professionally through their experiences. As project leaders, they develop core leadership skills, including team communication, delegation, intuition, and the ability to inspire others. They learn how to problem solve creatively and then translate their ideas and opinions into convincing arguments that gain stakeholder support. Students may also advance their personal technical skills such as computer programming or graphic design, which are increasingly in demand across the medical community.

As peer representatives, student members learn how to solicit and assimilate diverse perspectives and advocate on behalf of a group. Working closely with faculty offers them unique insight into the structure and politics of a large educational organization, which may inform their future work as teachers, leaders, and scholars in academia. Students also receive hands-on training and gain experience in conducting research in medical education, and may have opportunities to develop skills in scientific communication in written or oral formats. Most importantly, their experience may stimulate future learning and self-improvement around any of a number of topics in leadership, education, and technology.

## Discussion

### A Successful Model for Students as Stakeholders

Motivated by demonstrative examples of the benefits of integrated student involvement in advancing medical education technologies, we developed a new student-led technology committee model to inform effective use of these innovations. Thoughtful engagement of motivated students as stakeholders in the process of educational technology development and deployment was essential to our approach. Further, our STC model provided a basis for unifying efforts and goals of students and school leadership. Our experiences with the STC model have borne out that students, coupled with institutional drive, can help schools to realize technology-focused advancements in their educational ecology. Many opportunities are enabled by this model to generate immediate and long-term value for committee members, the student body, faculty, staff, and the school community.

We have gained the following key lessons, including mechanisms to address potential limitations of our approach, through the initial successes and challenges of implementing our model.

### Composing the Committee for Harmony

We learned the importance of the student composition of the committee to ensure alignment of interests and complementary skillsets. Whereas many student committees employ election processes based on popularity or first-come interest [23,24], such processes would likely be unreliable in selecting the best students for the STC model. Through a careful application and interview process, a select group of students is chosen to bring a mix of education and technology interests as well as technical and leadership skillsets (Table 2). Despite the technology-focused work of the committee, not all members need be technological experts. Recruiting such ideal students for the STC model may be challenging in the early days of a new committee. Student interest should be solicited by founding members and/or faculty advisors with recognition that students excited by education and/or informatics often do not know where to apply their interests without invitation.

### Fostering Student and Faculty Collaboration

We are fortunate at our institution to have an Assistant Dean for Educational Informatics and Technology to serve as faculty advisor and interface between the STC and the school leadership. Although more institutions are creating similar positions, schools without such dedicated roles should identify faculty advisors with experience in educational technology who are also strong communicators with constituents. As noted by others [23,24], we also found that it is essential for faculty and staff to have an open attitude toward student involvement and to affirm students’ contributions to institutional projects. As a 2-way street, there should also be clear support for STC initiatives from school leadership, offering connections to key faculty and staff, financial resources for appropriate projects, and an open door to provide feedback and guidance. Together, these practices empower students as change agents.

While involved faculty advisors and school leadership must make a significant commitment to fully support the STC model, we realized that they can benefit markedly from their participation. These individuals develop a keen appreciation for student input while also receiving gratification in knowing that they are advancing committee members’ and the student body’s experiences. They may also become co-authors on research publications led by the STC, helping to advance their own careers and contribute to the field of education.

### Identifying Appropriate Goals and Project Scope

In this paper, we have presented our framework of 4 core missions that guide committee initiatives. In addition, school leadership and committee members should regularly identify shared goals that can inform specific project targets. We also found that it is essential that salaried work (eg, technical support for students) be reserved only for faculty and staff.

The faculty advisor and committee leadership should collaborate to keep project scopes concise and focused, while simultaneously allowing members to dream big and wonder “What if...?”. There is potential concern that students do not have time to be involved in this level of work and may be distracted from their primary commitment as medical students. We have found that group accountability, shared project responsibilities, and input from the faculty advisor can help to keep members balanced and reduce such risks to students’ academic focus.

### Applying Our Lessons

Moving forward, the STC will need to develop measures of effectiveness to assess contributions to the school’s educational ecology. Although informal feedback can provide some guidance, routine surveys and objective metrics may elucidate opportunities to improve both the processes and outcomes of the STC model. These data may also enable the STC to secure additional funding for project development and pilots.

### Conclusion

As technology develops into an increasingly more essential component of medical education, students need to play a central role as stakeholders in the creation and refinement of medical education technologies. Through our novel student-led technology committee, active mentorship, and formalized, administrative commitment, we forged a collaborative vision and effort to effect positive change across 4 core missions. Students have demonstrated meaningful contributions to our institution’s education ecology while generating substantial benefits for committee members and faculty. Our experiences may serve as a model for other institutions to spark advancement of medical education technologies to improve training for 21st-century physicians.

## Abbreviations

IT: information technology
LMS: learning management system
STC: student technology committee
